# Bis[μ-1,3-bis­(di­phenyl­phosphan­yl)propane-κ^2^ *P*:*P*′]digold(I) tetra­chloridonickelate(II) diethyl ether monosolvate

**DOI:** 10.1107/S1600536813013470

**Published:** 2013-05-22

**Authors:** Asako Igashira-Kamiyama, Takuma Itai, Yuka Arai, Takumi Konno

**Affiliations:** a Department of Chemistry, Graduate School of Science, Osaka University, Toyonaka, Osaka 560-0043, Japan

## Abstract

The title compound, [Au_2_(C_27_H_26_P_2_)_2_][NiCl_4_]·C_4_H_10_O, consists of a digold(I) complex cation, an [NiCl_4_]^2−^ complex anion and a diethyl ether solvent mol­ecule. Two 1,3-bis­(di­phenyl­phosphan­yl)propane (dppp) ligands bridge two Au^I^ atoms, forming a metallacycle in which each of the Au^I^ atoms is coordinated in a slightly distorted linear environment by two P atoms. In the complex anion, the Ni^II^ atom is coordinated by four chloride ligands in a distorted tetra­hedral geometry. The complex cation and the complex anion form a cation–anion pair through two Au⋯Cl contacts of 3.040 (1) and 3.021 (2) Å. One of the phenyl groups of the dppp ligand is disordered over two positions with equal occupancies.

## Related literature

For closely related structures, see: Gruber & Jansen (2010 ▶); Brandys & Puddephatt (2001 ▶). For related studies, see: Igashira-Kamiyama *et al.* (2012 ▶); Lee *et al.* (2012 ▶); Lim *et al.* (2011 ▶); Hashimoto *et al.* (2010 ▶). For the starting material, see: Howard-Lock *et al.* (1986 ▶); Blondeau *et al.* (1967 ▶); Mirabelli *et al.* (1987 ▶). For a description of the Cambridge Structural Database, see: Allen (2002 ▶).
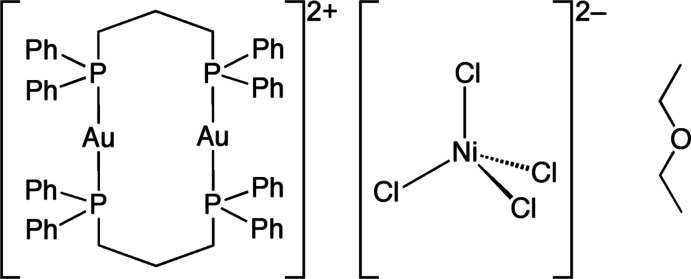


## Experimental

### 

#### Crystal data

[Au_2_(C_27_H_26_P_2_)_2_][NiCl_4_]·C_4_H_10_O
*M* *_r_* = 1493.40
Monoclinic, 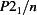

*a* = 18.9290 (5) Å
*b* = 16.1945 (7) Å
*c* = 19.0895 (17) Å
β = 97.368 (7)°
*V* = 5803.5 (6) Å^3^
*Z* = 4
Mo *K*α radiation
μ = 5.70 mm^−1^
*T* = 200 K
0.28 × 0.05 × 0.03 mm

#### Data collection

Rigaku R-AXIS VII diffractometer
Absorption correction: multi-scan (*ABSCOR*; Higashi, 1995 ▶) *T*_min_ = 0.580, *T*_max_ = 0.773
45449 measured reflections
13273 independent reflections
10666 reflections with *I* > 2σ(*I*)
*R*_int_ = 0.042

#### Refinement

*R*[*F* ^2^ > 2σ(*F* ^2^)] = 0.048
*wR*(*F* ^2^) = 0.083
*S* = 1.14
13273 reflections
685 parameters
78 restraints
H-atom parameters constrained
Δρ_max_ = 1.16 e Å^−3^
Δρ_min_ = −0.88 e Å^−3^

### 

Data collection: *PROCESS-AUTO* (Rigaku, 2000 ▶); cell refinement: *PROCESS-AUTO*; data reduction: *PROCESS-AUTO*; program(s) used to solve structure: *SHELXS97* (Sheldrick, 2008 ▶); program(s) used to refine structure: *SHELXL97* (Sheldrick, 2008 ▶); molecular graphics: *Yadokari-XG 2009* (Kabuto *et al.*, 2009 ▶); software used to prepare material for publication: *Yadokari-XG 2009*.

## Supplementary Material

Crystal structure: contains datablock(s) I, global. DOI: 10.1107/S1600536813013470/gk2571sup1.cif

Structure factors: contains datablock(s) I. DOI: 10.1107/S1600536813013470/gk2571Isup2.hkl

Additional supplementary materials:  crystallographic information; 3D view; checkCIF report

## Figures and Tables

**Table 1:** Selected bond lengths (Å)

Au1—P1	2.3109 (13)
Au1—P3	2.3129 (13)
Au2—P2	2.3013 (13)
Au2—P4	2.3050 (13)
Ni1—Cl4	2.2359 (15)
Ni1—Cl2	2.2548 (16)
Ni1—Cl1	2.2558 (15)
Ni1—Cl3	2.2780 (14)

**Fig. 1.**
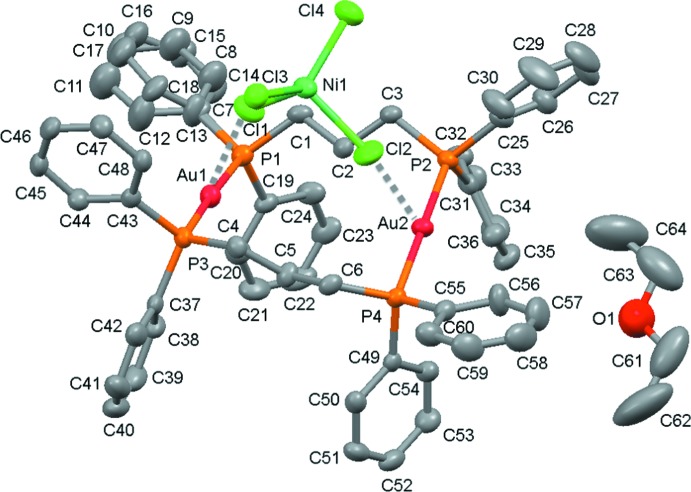
A view of the molecular structure of the title compound with the atom-numbering scheme. H atoms are omitted for clarity.

**Fig. 2.**
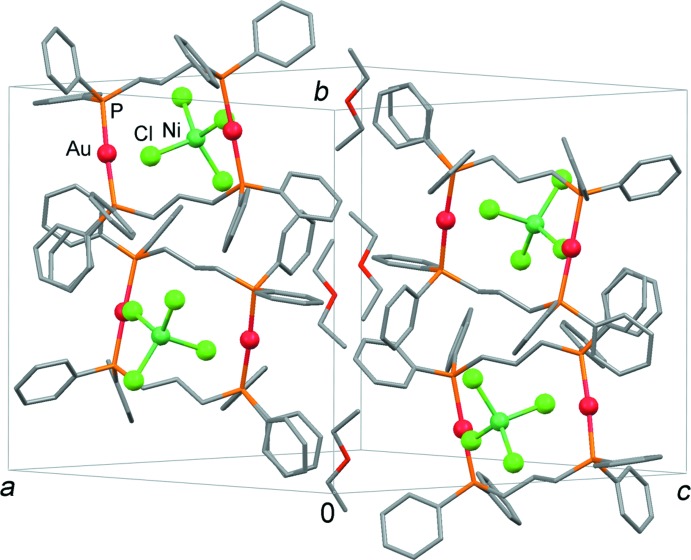
Crystal packing of the title compound. H atoms are omitted for clarity.

## supplementary crystallographic information

### Comment

In recent years, diphosphine-bridged cyclic digold(I) complexes have attracted considerable attention because of their intriguing photophysical properties that originate from the presence of an aurophilic interaction between gold(I) ions. A typical example is [Au_2_(dppm)_2_]^2+^ (dppm = bis(diphenylphosphanyl)methane), which is easily prepared from dppm having a methylene linker between two P atoms, in combination with an Au^I^ ion. Analogous digold(I) complexes having a longer linker between two P atoms, such as 1,2-bis(diphenylphosphanyl)ethane (dppe) and 1,3-bis(diphenylphosphanyl)propane (dppp), have also been prepared, but only a few of them have been structurally characterized to date (Allen, 2002).

Recently, we started to investigate the creation of diphosphine-bridged digold(I) metalloligands and their coordination behavior toward various metal ions, with the aim of the rational construction of heterometallic multinuclear and metallosupramolecular structures (Lee *et al.*, 2012; Igashira-Kamiyama *et al.*, 2012; Hashimoto *et al.*, 2010). In the course of this study, we found the formation of a diphosphine-bridged digold(I) complex, [Au_2_(dppp)_2_]^2+^ (dppp = 1,3-bis(diphenylphosphanyl)propane), which is cocrystallized with [NiCl_4_]^2–^ to give single-crystals of [Au_2_(dppp)_2_][NiCl_4_].Et_2_O (I). Here, we report the synthesis and crystal structure of (I).

Treatment of [Au_2_Cl_2_(dppp)] (Mirabelli *et al.*, 1987) with 1 equiv of *N*,*N'*-ethylene-bis-*D*-penicillamine (Howard-Lock *et al.*, 1986; Blondeau *et al.*, 1967) in ethanol/water in the presence of KOH gave a white powder. When this powder was reacted with NiCl_2_.6H_2_O in ethanol, blue needle crystals of [Au_2_(dppp)_2_][NiCl_4_].Et_2_O (I) were obtained by the vapor diffusion of diethylether into the reaction solution.

Compound (I) crystallized in a centrosymmetric space group *P*2_1_/*n*, which contains one digold(I) complex cation, one nickel(II) complex anion and one solvate diethylether molecule in the asymmetric unit. The complex cation has a cyclic digold(I) structure in [Au_2_(dppp)_2_]^2+^, in which two dppp ligands bridge two Au^I^ atoms. Each Au^I^ atom adopts a slightly distorted linear geometry (176.68 (5)°, 177.98 (5)°) coordinated by two P atoms from two different dppp ligands (Fig. 1). The Au–P bond distances (2.3013 (13)–2.3129 (13) Å) are within the range observed for [Au_2_(dppp)_2_]_2_[Mo_8_O_26_], [Au_2_(dppp)_2_]_2_[PMo_8_O_26_] and [Au_2_(dppp)_2_](CF_3_CO_2_)_2_ (Au–P = 2.271–2.324 Å) (Gruber *et al.*, 2010; Brandys *et al.*, 2001). In the complex-cation of (I), two Au^I^ atoms and four P atoms are deviated from co-planarity, with the dihedral angle between two Au_2_P_2_ planes being 21.94 (2)°. This structural feature is different from those found in [Au_2_(dppp)_2_]_2_[Mo_8_O_26_], [Au_2_(dppp)_2_]_2_[PMo_8_O_26_] and [Au_2_(dppp)_2_](CF_3_CO_2_)_2_, in which all the Au^I^ and P atoms in each complex-cation are located nearly on the same plane (Gruber *et al.*, 2010; Brandys *et al.*, 2001). The distance between two Au^I^ atoms in [Au_2_(dppp)_2_]^2+^ is 5.5424 (4) Å, indicative of the absence of an aurophilic interaction. In (I), a nickel(II) complex-anion, [NiCl_4_]^2–^, which has a distorted tetrahedral geometry, coexists with [Au_2_(dppp)_2_]^2+^ to balance the total charge. Note that [Au_2_(dppp)_2_]^2+^ and [NiCl_4_]^2–^ are connected to each other through two Au···Cl bonding interactions (3.040 (1) Å and 3.021 (2) Å), while any other significant intermolecular interactions do not exist in (I) (Fig. 2).

### Experimental

To a white suspension containing 0.15 g (0.17 mmol) of [Au_2_Cl_2_(dppp)] in 300 ml of EtOH was added 0.056 g (0.17 mmol) of *N*,*N'*-ethylene-bis-*D*-penicillamine dissolved in 3 ml of a 0.1 *M* KOH aqueous solution. The mixture was stirred at room temperature for 3 h. The resulting colorless solution was evaporated to dryness, and then the residue was washed with H_2_O to give a white powder (0.15 g). When 2.5 mg (0.01 mmol) of NiCl_2_.6H_2_O was added to a suspension of this white powder (10 mg) in 1 ml of EtOH, followed by stirring at room temperature for 3 h, a clear red orange solution was obtained. Vapor diffusion of diethylether into the resulting red orange solution afforded a small amount of blue needle crystals of [Au_2_(dppp)_2_][NiCl_4_].Et_2_O.

### Refinement

H atoms were placed at calculated positions [C–H = 0.99 (methylene) or 0.95 Å (phenyl)] and refined as riding with *U*_iso_(H) = 1.2*U*_eq_(C). One phenyl ring is disordered over two positions (C7–C12 and C13–C18) with site occupancies of 0.5. The FLAT and SIMU restraints were used to model the disordered phenyl ring.

### Crystal data

**Table:**

[Au_2_(C_27_H_26_P_2_)_2_][NiCl_4_]·C_4_H_10_O	*F*(000) = 2928
*M**_r_* = 1493.40	*D*_x_ = 1.709 Mg m^−^^3^
Monoclinic, *P*2_1_/*n*	Mo *K*α radiation, λ = 0.71075 Å
Hall symbol: -P 2yn	Cell parameters from 2299 reflections
*a* = 18.9290 (5) Å	θ = 4.1–27.6°
*b* = 16.1945 (7) Å	µ = 5.70 mm^−^^1^
*c* = 19.0895 (17) Å	*T* = 200 K
β = 97.368 (7)°	Needle, blue
*V* = 5803.5 (6) Å^3^	0.28 × 0.05 × 0.03 mm
*Z* = 4	

### Data collection

**Table:**

Rigaku R-AXIS VII diffractometer	13273 independent reflections
Radiation source: fine-focus sealed tube	10666 reflections with *I* > 2σ(*I*)
Graphite monochromator	*R*_int_ = 0.042
Detector resolution: 10.000 pixels mm^-1^	θ_max_ = 27.5°, θ_min_ = 3.0°
ω scans	*h* = −22→24
Absorption correction: multi-scan (*ABSCOR*; Higashi, 1995)	*k* = −20→21
*T*_min_ = 0.580, *T*_max_ = 0.773	*l* = −24→24
45449 measured reflections	

### Refinement

**Table:**

Refinement on *F*^2^	Primary atom site location: structure-invariant direct methods
Least-squares matrix: full	Secondary atom site location: difference Fourier map
*R*[*F*^2^ > 2σ(*F*^2^)] = 0.048	Hydrogen site location: inferred from neighbouring sites
*wR*(*F*^2^) = 0.083	H-atom parameters constrained
*S* = 1.14	*w* = 1/[σ^2^(*F*_o_^2^) + (0.0332*P*)^2^ + 5.0632*P*] where *P* = (*F*_o_^2^ + 2*F*_c_^2^)/3
13273 reflections	(Δ/σ)_max_ = 0.001
685 parameters	Δρ_max_ = 1.16 e Å^−^^3^
78 restraints	Δρ_min_ = −0.88 e Å^−^^3^

### Special details

**Table:**

Geometry. All e.s.d.'s (except the e.s.d. in the dihedral angle between two l.s. planes) are estimated using the full covariance matrix. The cell e.s.d.'s are taken into account individually in the estimation of e.s.d.'s in distances, angles and torsion angles; correlations between e.s.d.'s in cell parameters are only used when they are defined by crystal symmetry. An approximate (isotropic) treatment of cell e.s.d.'s is used for estimating e.s.d.'s involving l.s. planes.
Refinement. Refinement of *F*^2^ against ALL reflections. The weighted *R*-factor w*R* and goodness of fit *S* are based on *F*^2^, conventional *R*-factors *R* are based on *F*, with *F* set to zero for negative *F*^2^. The threshold expression of *F*^2^ > σ(*F*^2^) is used only for calculating *R*-factors(gt) *etc*. and is not relevant to the choice of reflections for refinement. *R*-factors based on *F*^2^ are statistically about twice as large as those based on *F*, and *R*-factors based on ALL data will be even larger.

### Fractional atomic coordinates and isotropic or equivalent isotropic displacement parameters (Å^2^)

**Table:**

	*x*	*y*	*z*	*U*_iso_*/*U*_eq_	Occ. (<1)
Au1	0.779915 (10)	0.834674 (12)	0.074536 (10)	0.03063 (6)	
Au2	0.549659 (10)	0.900739 (12)	0.218117 (10)	0.02907 (6)	
P1	0.73666 (7)	0.70192 (8)	0.05633 (7)	0.0304 (3)	
P2	0.53747 (7)	0.76109 (8)	0.23538 (7)	0.0285 (3)	
P3	0.81787 (6)	0.96902 (8)	0.09657 (7)	0.0271 (3)	
P4	0.56077 (6)	1.03989 (8)	0.19661 (7)	0.0266 (3)	
C1	0.7040 (3)	0.6692 (3)	0.1377 (3)	0.0324 (12)	
H1	0.6837	0.6129	0.1313	0.039*	
H2	0.7443	0.6670	0.1762	0.039*	
C2	0.6472 (2)	0.7282 (3)	0.1583 (3)	0.0306 (11)	
H3	0.6057	0.7270	0.1210	0.037*	
H4	0.6666	0.7850	0.1605	0.037*	
C3	0.6218 (2)	0.7075 (3)	0.2294 (2)	0.0296 (11)	
H5	0.6580	0.7251	0.2686	0.036*	
H6	0.6149	0.6471	0.2333	0.036*	
C4	0.7729 (2)	1.0247 (3)	0.1619 (3)	0.0289 (11)	
H7	0.7878	1.0008	0.2092	0.035*	
H8	0.7881	1.0832	0.1630	0.035*	
C5	0.6909 (2)	1.0206 (3)	0.1457 (3)	0.0348 (12)	
H9	0.6760	1.0424	0.0976	0.042*	
H10	0.6754	0.9622	0.1466	0.042*	
C6	0.6541 (2)	1.0700 (3)	0.1987 (3)	0.0304 (11)	
H11	0.6568	1.1296	0.1877	0.036*	
H12	0.6794	1.0610	0.2468	0.036*	
C7	0.802 (2)	0.623 (3)	0.0354 (17)	0.055 (4)	0.50
C8	0.7861 (11)	0.5432 (14)	0.0384 (9)	0.064 (4)	0.50
H13	0.7424	0.5264	0.0537	0.077*	0.50
C9	0.8353 (12)	0.4825 (17)	0.0181 (13)	0.070 (4)	0.50
H14	0.8255	0.4253	0.0218	0.085*	0.50
C10	0.8946 (13)	0.5076 (15)	−0.0057 (11)	0.075 (4)	0.50
H15	0.9253	0.4681	−0.0228	0.090*	0.50
C11	0.9112 (9)	0.5852 (10)	−0.0060 (10)	0.078 (3)	0.50
H16	0.9559	0.6015	−0.0191	0.094*	0.50
C12	0.8637 (8)	0.6454 (10)	0.0129 (9)	0.067 (3)	0.50
H17	0.8751	0.7023	0.0097	0.081*	0.50
C13	0.7966 (16)	0.622 (3)	0.0378 (12)	0.030 (3)	0.50
C14	0.7708 (9)	0.5416 (12)	0.0162 (8)	0.042 (3)	0.50
H18	0.7210	0.5317	0.0094	0.051*	0.50
C15	0.8156 (8)	0.4794 (15)	0.0055 (11)	0.049 (3)	0.50
H19	0.7983	0.4265	−0.0099	0.059*	0.50
C16	0.8907 (12)	0.4968 (14)	0.0186 (9)	0.056 (3)	0.50
H20	0.9236	0.4548	0.0101	0.067*	0.50
C17	0.9153 (7)	0.5694 (9)	0.0421 (8)	0.056 (3)	0.50
H21	0.9652	0.5785	0.0516	0.068*	0.50
C18	0.8683 (7)	0.6319 (9)	0.0526 (7)	0.045 (3)	0.50
H22	0.8866	0.6833	0.0708	0.054*	0.50
C19	0.6622 (3)	0.6947 (3)	−0.0140 (3)	0.0304 (11)	
C20	0.6632 (3)	0.7447 (3)	−0.0737 (3)	0.0430 (14)	
H23	0.7014	0.7821	−0.0764	0.052*	
C21	0.6076 (3)	0.7395 (4)	−0.1294 (3)	0.0500 (16)	
H24	0.6087	0.7727	−0.1703	0.060*	
C22	0.5512 (3)	0.6863 (4)	−0.1253 (3)	0.0525 (17)	
H25	0.5130	0.6839	−0.1627	0.063*	
C23	0.5506 (3)	0.6369 (3)	−0.0668 (3)	0.0469 (15)	
H26	0.5123	0.5996	−0.0647	0.056*	
C24	0.6050 (3)	0.6407 (3)	−0.0109 (3)	0.0404 (13)	
H27	0.6034	0.6067	0.0294	0.048*	
C25	0.5129 (3)	0.7377 (3)	0.3220 (3)	0.0354 (12)	
C26	0.4619 (3)	0.6787 (3)	0.3322 (3)	0.0456 (15)	
H28	0.4399	0.6472	0.2934	0.055*	
C27	0.4431 (4)	0.6658 (4)	0.3999 (4)	0.0574 (18)	
H29	0.4071	0.6269	0.4069	0.069*	
C28	0.4769 (4)	0.7097 (4)	0.4565 (4)	0.072 (2)	
H30	0.4649	0.7002	0.5026	0.086*	
C29	0.5281 (5)	0.7671 (4)	0.4462 (3)	0.078 (2)	
H31	0.5512	0.7973	0.4852	0.093*	
C30	0.5462 (4)	0.7811 (4)	0.3794 (3)	0.0577 (18)	
H32	0.5816	0.8209	0.3728	0.069*	
C31	0.4727 (2)	0.7128 (3)	0.1696 (3)	0.0279 (11)	
C32	0.4813 (3)	0.6337 (3)	0.1438 (3)	0.0432 (14)	
H33	0.5202	0.6007	0.1638	0.052*	
C33	0.4338 (3)	0.6028 (4)	0.0894 (3)	0.0498 (15)	
H34	0.4401	0.5483	0.0728	0.060*	
C34	0.3779 (3)	0.6490 (4)	0.0588 (3)	0.0518 (16)	
H35	0.3464	0.6274	0.0204	0.062*	
C35	0.3675 (3)	0.7272 (4)	0.0841 (3)	0.0485 (15)	
H36	0.3277	0.7591	0.0644	0.058*	
C36	0.4150 (3)	0.7590 (3)	0.1381 (3)	0.0366 (12)	
H37	0.4083	0.8135	0.1542	0.044*	
C37	0.8062 (2)	1.0336 (3)	0.0176 (3)	0.0303 (11)	
C38	0.7747 (3)	1.0013 (4)	−0.0457 (3)	0.0444 (14)	
H38	0.7611	0.9448	−0.0481	0.053*	
C39	0.7627 (3)	1.0501 (5)	−0.1058 (3)	0.0565 (18)	
H39	0.7405	1.0277	−0.1491	0.068*	
C40	0.7836 (3)	1.1317 (5)	−0.1014 (4)	0.0618 (19)	
H40	0.7763	1.1655	−0.1423	0.074*	
C41	0.8145 (3)	1.1645 (4)	−0.0391 (4)	0.0562 (18)	
H41	0.8276	1.2211	−0.0367	0.067*	
C42	0.8268 (3)	1.1151 (3)	0.0208 (3)	0.0422 (14)	
H42	0.8495	1.1377	0.0639	0.051*	
C43	0.9125 (2)	0.9708 (3)	0.1293 (3)	0.0269 (11)	
C44	0.9617 (3)	0.9585 (3)	0.0813 (3)	0.0345 (12)	
H43	0.9457	0.9566	0.0320	0.041*	
C45	1.0339 (3)	0.9489 (3)	0.1052 (3)	0.0396 (13)	
H44	1.0666	0.9401	0.0721	0.048*	
C46	1.0583 (3)	0.9520 (3)	0.1762 (3)	0.0377 (13)	
H45	1.1076	0.9455	0.1920	0.045*	
C47	1.0108 (3)	0.9648 (3)	0.2241 (3)	0.0363 (12)	
H46	1.0276	0.9679	0.2731	0.044*	
C48	0.9377 (3)	0.9733 (3)	0.2010 (3)	0.0318 (11)	
H47	0.9052	0.9809	0.2345	0.038*	
C49	0.5141 (2)	1.0717 (3)	0.1118 (2)	0.0265 (10)	
C50	0.5378 (3)	1.1376 (3)	0.0733 (3)	0.0341 (12)	
H48	0.5804	1.1664	0.0900	0.041*	
C51	0.4972 (3)	1.1601 (3)	0.0095 (3)	0.0434 (14)	
H49	0.5123	1.2050	−0.0170	0.052*	
C52	0.4360 (3)	1.1183 (4)	−0.0153 (3)	0.0420 (14)	
H50	0.4093	1.1340	−0.0588	0.050*	
C53	0.4130 (3)	1.0534 (3)	0.0231 (3)	0.0409 (14)	
H51	0.3702	1.0251	0.0064	0.049*	
C54	0.4522 (3)	1.0298 (3)	0.0853 (3)	0.0343 (12)	
H52	0.4368	0.9842	0.1108	0.041*	
C55	0.5201 (3)	1.1036 (3)	0.2596 (3)	0.0320 (12)	
C56	0.5434 (3)	1.1835 (3)	0.2746 (3)	0.0463 (15)	
H53	0.5833	1.2046	0.2549	0.056*	
C57	0.5084 (4)	1.2322 (4)	0.3184 (3)	0.0583 (18)	
H54	0.5246	1.2869	0.3289	0.070*	
C58	0.4510 (4)	1.2029 (5)	0.3467 (4)	0.067 (2)	
H55	0.4272	1.2376	0.3763	0.081*	
C59	0.4271 (4)	1.1241 (5)	0.3329 (4)	0.069 (2)	
H56	0.3869	1.1040	0.3528	0.083*	
C60	0.4624 (3)	1.0732 (4)	0.2892 (3)	0.0530 (16)	
H57	0.4468	1.0181	0.2800	0.064*	
Ni1	0.79494 (3)	0.85982 (4)	0.34081 (3)	0.03311 (16)	
Cl1	0.81798 (9)	0.81747 (9)	0.23347 (7)	0.0496 (4)	
Cl2	0.68553 (8)	0.91672 (11)	0.32021 (9)	0.0624 (5)	
Cl3	0.87604 (7)	0.96027 (8)	0.37596 (7)	0.0377 (3)	
Cl4	0.77899 (8)	0.74535 (9)	0.40236 (8)	0.0508 (4)	
O1	0.2306 (3)	0.4928 (4)	0.2151 (3)	0.0986 (18)	
C61	0.2757 (5)	0.5546 (8)	0.2001 (6)	0.125 (4)	
H58	0.2643	0.5702	0.1498	0.150*	
H59	0.3252	0.5337	0.2072	0.150*	
C62	0.2710 (6)	0.6288 (8)	0.2443 (8)	0.171 (6)	
H60	0.3041	0.6709	0.2312	0.257*	
H61	0.2835	0.6142	0.2942	0.257*	
H62	0.2223	0.6505	0.2369	0.257*	
C63	0.2334 (7)	0.4189 (7)	0.1756 (5)	0.125 (4)	
H63	0.2798	0.3912	0.1889	0.151*	
H64	0.2286	0.4319	0.1246	0.151*	
C64	0.1743 (8)	0.3632 (7)	0.1905 (7)	0.174 (6)	
H65	0.1764	0.3118	0.1637	0.260*	
H66	0.1285	0.3905	0.1764	0.260*	
H67	0.1793	0.3507	0.2411	0.260*	

### Atomic displacement parameters (Å^2^)

**Table:**

	*U*^11^	*U*^22^	*U*^33^	*U*^12^	*U*^13^	*U*^23^
Au1	0.02931 (11)	0.03070 (12)	0.03187 (11)	−0.00375 (8)	0.00394 (8)	−0.00447 (8)
Au2	0.03258 (11)	0.02486 (11)	0.02932 (11)	0.00187 (8)	0.00220 (8)	0.00207 (8)
P1	0.0302 (7)	0.0293 (7)	0.0310 (7)	−0.0001 (6)	0.0014 (6)	−0.0036 (6)
P2	0.0322 (7)	0.0262 (7)	0.0271 (7)	0.0005 (6)	0.0038 (5)	0.0034 (5)
P3	0.0232 (7)	0.0300 (7)	0.0283 (7)	−0.0020 (5)	0.0043 (5)	−0.0013 (5)
P4	0.0250 (7)	0.0248 (7)	0.0297 (7)	0.0018 (5)	0.0029 (5)	−0.0008 (5)
C1	0.038 (3)	0.023 (3)	0.034 (3)	0.003 (2)	−0.004 (2)	0.002 (2)
C2	0.026 (3)	0.029 (3)	0.036 (3)	0.000 (2)	0.003 (2)	0.002 (2)
C3	0.031 (3)	0.026 (3)	0.031 (3)	0.004 (2)	0.000 (2)	0.004 (2)
C4	0.029 (3)	0.028 (3)	0.029 (3)	−0.005 (2)	0.004 (2)	−0.003 (2)
C5	0.027 (3)	0.038 (3)	0.039 (3)	−0.001 (2)	0.002 (2)	−0.009 (2)
C6	0.032 (3)	0.027 (3)	0.031 (3)	0.007 (2)	0.000 (2)	−0.007 (2)
C7	0.046 (8)	0.048 (7)	0.077 (8)	0.005 (6)	0.026 (6)	−0.011 (7)
C8	0.057 (8)	0.055 (7)	0.084 (8)	0.013 (6)	0.019 (6)	−0.008 (7)
C9	0.063 (8)	0.054 (7)	0.098 (9)	0.015 (7)	0.020 (7)	−0.007 (7)
C10	0.065 (7)	0.061 (7)	0.104 (9)	0.019 (6)	0.026 (7)	−0.011 (7)
C11	0.063 (7)	0.064 (7)	0.111 (8)	0.004 (6)	0.023 (7)	−0.009 (7)
C12	0.051 (7)	0.055 (7)	0.100 (8)	−0.002 (5)	0.026 (6)	−0.012 (6)
C13	0.023 (6)	0.036 (6)	0.030 (5)	0.003 (5)	−0.005 (5)	−0.006 (5)
C14	0.030 (6)	0.042 (6)	0.053 (6)	0.001 (5)	−0.001 (5)	−0.010 (5)
C15	0.035 (6)	0.044 (6)	0.067 (7)	0.006 (5)	0.005 (5)	−0.003 (5)
C16	0.040 (6)	0.057 (7)	0.072 (7)	0.014 (5)	0.012 (6)	0.005 (6)
C17	0.034 (5)	0.067 (6)	0.068 (6)	0.009 (5)	0.006 (5)	−0.002 (5)
C18	0.030 (5)	0.052 (6)	0.052 (5)	0.010 (4)	−0.003 (5)	−0.008 (5)
C19	0.030 (3)	0.030 (3)	0.029 (3)	0.007 (2)	−0.003 (2)	−0.005 (2)
C20	0.047 (3)	0.040 (3)	0.043 (3)	0.002 (3)	0.008 (3)	−0.002 (3)
C21	0.064 (4)	0.056 (4)	0.028 (3)	0.014 (3)	−0.003 (3)	0.001 (3)
C22	0.058 (4)	0.050 (4)	0.043 (4)	0.017 (3)	−0.021 (3)	−0.015 (3)
C23	0.039 (3)	0.034 (3)	0.063 (4)	−0.003 (3)	−0.010 (3)	−0.013 (3)
C24	0.045 (3)	0.026 (3)	0.047 (3)	−0.005 (2)	−0.007 (3)	−0.002 (2)
C25	0.048 (3)	0.029 (3)	0.031 (3)	0.007 (2)	0.010 (2)	0.007 (2)
C26	0.054 (4)	0.042 (4)	0.043 (3)	−0.001 (3)	0.015 (3)	0.004 (3)
C27	0.078 (5)	0.043 (4)	0.059 (4)	−0.001 (3)	0.036 (4)	0.010 (3)
C28	0.129 (7)	0.053 (4)	0.040 (4)	0.007 (5)	0.038 (4)	0.005 (3)
C29	0.138 (7)	0.066 (5)	0.029 (4)	−0.020 (5)	0.009 (4)	−0.004 (3)
C30	0.087 (5)	0.051 (4)	0.035 (3)	−0.019 (4)	0.006 (3)	0.003 (3)
C31	0.026 (3)	0.027 (3)	0.032 (3)	−0.003 (2)	0.009 (2)	0.002 (2)
C32	0.046 (3)	0.032 (3)	0.049 (4)	−0.002 (3)	−0.005 (3)	0.002 (3)
C33	0.052 (4)	0.035 (3)	0.061 (4)	−0.005 (3)	0.003 (3)	−0.010 (3)
C34	0.048 (4)	0.061 (4)	0.043 (4)	−0.009 (3)	−0.004 (3)	−0.006 (3)
C35	0.034 (3)	0.062 (4)	0.048 (4)	0.009 (3)	−0.003 (3)	−0.002 (3)
C36	0.035 (3)	0.038 (3)	0.037 (3)	0.004 (2)	0.002 (2)	−0.004 (2)
C37	0.023 (3)	0.037 (3)	0.033 (3)	0.006 (2)	0.011 (2)	0.005 (2)
C38	0.034 (3)	0.064 (4)	0.034 (3)	0.003 (3)	0.002 (2)	0.009 (3)
C39	0.043 (4)	0.092 (6)	0.035 (3)	0.016 (4)	0.006 (3)	0.010 (3)
C40	0.058 (4)	0.077 (5)	0.053 (4)	0.034 (4)	0.018 (3)	0.030 (4)
C41	0.063 (4)	0.049 (4)	0.062 (4)	0.019 (3)	0.028 (4)	0.020 (3)
C42	0.043 (3)	0.042 (4)	0.045 (3)	0.013 (3)	0.019 (3)	0.004 (3)
C43	0.028 (3)	0.019 (3)	0.033 (3)	0.001 (2)	0.005 (2)	−0.002 (2)
C44	0.035 (3)	0.040 (3)	0.029 (3)	0.004 (2)	0.005 (2)	0.001 (2)
C45	0.030 (3)	0.044 (3)	0.047 (3)	0.005 (2)	0.016 (3)	0.008 (3)
C46	0.023 (3)	0.038 (3)	0.051 (4)	0.000 (2)	0.003 (2)	0.004 (3)
C47	0.035 (3)	0.039 (3)	0.034 (3)	−0.004 (2)	0.002 (2)	−0.001 (2)
C48	0.030 (3)	0.030 (3)	0.037 (3)	0.000 (2)	0.010 (2)	0.000 (2)
C49	0.025 (3)	0.027 (3)	0.029 (3)	0.006 (2)	0.008 (2)	−0.001 (2)
C50	0.035 (3)	0.027 (3)	0.042 (3)	0.003 (2)	0.011 (2)	0.002 (2)
C51	0.056 (4)	0.039 (3)	0.036 (3)	0.014 (3)	0.012 (3)	0.012 (3)
C52	0.041 (3)	0.052 (4)	0.032 (3)	0.021 (3)	0.001 (3)	−0.002 (3)
C53	0.039 (3)	0.046 (4)	0.036 (3)	0.001 (3)	−0.003 (3)	−0.006 (3)
C54	0.033 (3)	0.031 (3)	0.039 (3)	0.002 (2)	0.003 (2)	0.003 (2)
C55	0.034 (3)	0.035 (3)	0.028 (3)	0.008 (2)	0.005 (2)	−0.002 (2)
C56	0.062 (4)	0.037 (4)	0.041 (3)	0.009 (3)	0.013 (3)	0.000 (3)
C57	0.087 (5)	0.043 (4)	0.048 (4)	0.014 (4)	0.017 (4)	−0.008 (3)
C58	0.089 (6)	0.067 (5)	0.051 (4)	0.036 (4)	0.027 (4)	−0.002 (4)
C59	0.066 (5)	0.084 (6)	0.065 (5)	0.013 (4)	0.037 (4)	0.002 (4)
C60	0.051 (4)	0.051 (4)	0.063 (4)	−0.003 (3)	0.031 (3)	−0.005 (3)
Ni1	0.0343 (4)	0.0337 (4)	0.0296 (4)	−0.0045 (3)	−0.0027 (3)	0.0019 (3)
Cl1	0.0703 (10)	0.0470 (9)	0.0309 (7)	−0.0074 (7)	0.0042 (7)	−0.0045 (6)
Cl2	0.0373 (8)	0.0790 (12)	0.0660 (11)	0.0071 (8)	−0.0121 (7)	0.0174 (9)
Cl3	0.0343 (7)	0.0340 (7)	0.0437 (8)	−0.0041 (6)	0.0011 (6)	−0.0060 (6)
Cl4	0.0627 (10)	0.0424 (9)	0.0474 (9)	−0.0060 (7)	0.0077 (7)	0.0110 (7)
O1	0.106 (5)	0.114 (5)	0.080 (4)	−0.022 (4)	0.031 (4)	−0.001 (4)
C61	0.063 (6)	0.172 (11)	0.138 (10)	−0.017 (7)	0.002 (6)	0.068 (9)
C62	0.128 (10)	0.147 (11)	0.220 (16)	−0.082 (9)	−0.050 (10)	0.032 (11)
C63	0.191 (12)	0.114 (9)	0.075 (7)	0.044 (8)	0.029 (7)	−0.010 (6)
C64	0.281 (18)	0.105 (9)	0.134 (11)	−0.074 (11)	0.023 (11)	−0.022 (8)

### Geometric parameters (Å, º)

**Table:**

Au1—P1	2.3109 (13)	C29—H31	0.9500
Au1—P3	2.3129 (13)	C30—H32	0.9500
Au2—P2	2.3013 (13)	C31—C32	1.389 (7)
Au2—P4	2.3050 (13)	C31—C36	1.395 (7)
P1—C13	1.78 (4)	C32—C33	1.378 (7)
P1—C19	1.820 (5)	C32—H33	0.9500
P1—C1	1.822 (5)	C33—C34	1.364 (8)
P1—C7	1.87 (5)	C33—H34	0.9500
P2—C25	1.815 (5)	C34—C35	1.378 (8)
P2—C31	1.816 (5)	C34—H35	0.9500
P2—C3	1.833 (5)	C35—C36	1.377 (7)
P3—C43	1.820 (5)	C35—H36	0.9500
P3—C37	1.824 (5)	C36—H37	0.9500
P3—C4	1.834 (5)	C37—C42	1.377 (7)
P4—C49	1.816 (5)	C37—C38	1.379 (7)
P4—C6	1.828 (5)	C38—C39	1.388 (8)
P4—C55	1.829 (5)	C38—H38	0.9500
C1—C2	1.528 (7)	C39—C40	1.378 (9)
C1—H1	0.9900	C39—H39	0.9500
C1—H2	0.9900	C40—C41	1.363 (9)
C2—C3	1.535 (6)	C40—H40	0.9500
C2—H3	0.9900	C41—C42	1.390 (8)
C2—H4	0.9900	C41—H41	0.9500
C3—H5	0.9900	C42—H42	0.9500
C3—H6	0.9900	C43—C48	1.391 (7)
C4—C5	1.545 (6)	C43—C44	1.402 (7)
C4—H7	0.9900	C44—C45	1.393 (7)
C4—H8	0.9900	C44—H43	0.9500
C5—C6	1.526 (6)	C45—C46	1.375 (7)
C5—H9	0.9900	C45—H44	0.9500
C5—H10	0.9900	C46—C47	1.379 (7)
C6—H11	0.9900	C46—H45	0.9500
C6—H12	0.9900	C47—C48	1.405 (7)
C7—C8	1.33 (5)	C47—H46	0.9500
C7—C12	1.34 (4)	C48—H47	0.9500
C8—C9	1.44 (3)	C49—C54	1.392 (7)
C8—H13	0.9500	C49—C50	1.403 (7)
C9—C10	1.33 (3)	C50—C51	1.402 (7)
C9—H14	0.9500	C50—H48	0.9500
C10—C11	1.30 (3)	C51—C52	1.373 (8)
C10—H15	0.9500	C51—H49	0.9500
C11—C12	1.40 (2)	C52—C53	1.383 (8)
C11—H16	0.9500	C52—H50	0.9500
C12—H17	0.9500	C53—C54	1.372 (7)
C13—C18	1.36 (3)	C53—H51	0.9500
C13—C14	1.44 (4)	C54—H52	0.9500
C14—C15	1.35 (2)	C55—C60	1.383 (7)
C14—H18	0.9500	C55—C56	1.384 (7)
C15—C16	1.44 (3)	C56—C57	1.379 (8)
C15—H19	0.9500	C56—H53	0.9500
C16—C17	1.32 (2)	C57—C58	1.359 (9)
C16—H20	0.9500	C57—H54	0.9500
C17—C18	1.380 (17)	C58—C59	1.368 (10)
C17—H21	0.9500	C58—H55	0.9500
C18—H22	0.9500	C59—C60	1.401 (8)
C19—C24	1.400 (7)	C59—H56	0.9500
C19—C20	1.401 (7)	C60—H57	0.9500
C20—C21	1.399 (7)	Ni1—Cl4	2.2359 (15)
C20—H23	0.9500	Ni1—Cl2	2.2548 (16)
C21—C22	1.382 (8)	Ni1—Cl1	2.2558 (15)
C21—H24	0.9500	Ni1—Cl3	2.2780 (14)
C22—C23	1.375 (8)	O1—C61	1.370 (10)
C22—H25	0.9500	O1—C63	1.418 (10)
C23—C24	1.386 (7)	C61—C62	1.477 (15)
C23—H26	0.9500	C61—H58	0.9900
C24—H27	0.9500	C61—H59	0.9900
C25—C30	1.384 (7)	C62—H60	0.9800
C25—C26	1.390 (7)	C62—H61	0.9800
C26—C27	1.399 (8)	C62—H62	0.9800
C26—H28	0.9500	C63—C64	1.492 (14)
C27—C28	1.380 (9)	C63—H63	0.9900
C27—H29	0.9500	C63—H64	0.9900
C28—C29	1.375 (9)	C64—H65	0.9800
C28—H30	0.9500	C64—H66	0.9800
C29—C30	1.379 (8)	C64—H67	0.9800
			
P1—Au1—P3	176.68 (5)	C29—C28—H30	120.0
P2—Au2—P4	177.98 (5)	C27—C28—H30	120.0
C13—P1—C19	105.0 (8)	C28—C29—C30	120.4 (6)
C13—P1—C1	104.7 (10)	C28—C29—H31	119.8
C19—P1—C1	107.1 (2)	C30—C29—H31	119.8
C19—P1—C7	105.8 (11)	C29—C30—C25	120.4 (6)
C1—P1—C7	106.9 (12)	C29—C30—H32	119.8
C13—P1—Au1	118.7 (11)	C25—C30—H32	119.8
C19—P1—Au1	113.35 (17)	C32—C31—C36	117.5 (5)
C1—P1—Au1	107.16 (16)	C32—C31—P2	122.9 (4)
C7—P1—Au1	116.0 (14)	C36—C31—P2	119.3 (4)
C25—P2—C31	108.1 (2)	C33—C32—C31	120.5 (5)
C25—P2—C3	106.5 (2)	C33—C32—H33	119.8
C31—P2—C3	105.6 (2)	C31—C32—H33	119.8
C25—P2—Au2	112.19 (17)	C34—C33—C32	121.2 (6)
C31—P2—Au2	113.28 (16)	C34—C33—H34	119.4
C3—P2—Au2	110.65 (16)	C32—C33—H34	119.4
C43—P3—C37	106.5 (2)	C33—C34—C35	119.5 (6)
C43—P3—C4	106.4 (2)	C33—C34—H35	120.2
C37—P3—C4	105.2 (2)	C35—C34—H35	120.2
C43—P3—Au1	110.24 (15)	C36—C35—C34	119.7 (5)
C37—P3—Au1	112.76 (18)	C36—C35—H36	120.1
C4—P3—Au1	115.17 (16)	C34—C35—H36	120.1
C49—P4—C6	107.6 (2)	C35—C36—C31	121.5 (5)
C49—P4—C55	103.2 (2)	C35—C36—H37	119.2
C6—P4—C55	108.8 (2)	C31—C36—H37	119.2
C49—P4—Au2	112.99 (16)	C42—C37—C38	119.4 (5)
C6—P4—Au2	111.52 (16)	C42—C37—P3	120.6 (4)
C55—P4—Au2	112.28 (17)	C38—C37—P3	119.9 (4)
C2—C1—P1	111.4 (3)	C37—C38—C39	121.0 (6)
C2—C1—H1	109.4	C37—C38—H38	119.5
P1—C1—H1	109.4	C39—C38—H38	119.5
C2—C1—H2	109.4	C40—C39—C38	118.7 (6)
P1—C1—H2	109.4	C40—C39—H39	120.6
H1—C1—H2	108.0	C38—C39—H39	120.6
C1—C2—C3	113.7 (4)	C41—C40—C39	120.9 (6)
C1—C2—H3	108.8	C41—C40—H40	119.5
C3—C2—H3	108.8	C39—C40—H40	119.5
C1—C2—H4	108.8	C40—C41—C42	120.1 (6)
C3—C2—H4	108.8	C40—C41—H41	120.0
H3—C2—H4	107.7	C42—C41—H41	120.0
C2—C3—P2	109.0 (3)	C37—C42—C41	119.9 (6)
C2—C3—H5	109.9	C37—C42—H42	120.1
P2—C3—H5	109.9	C41—C42—H42	120.1
C2—C3—H6	109.9	C48—C43—C44	118.4 (4)
P2—C3—H6	109.9	C48—C43—P3	122.4 (4)
H5—C3—H6	108.3	C44—C43—P3	118.7 (4)
C5—C4—P3	112.7 (3)	C45—C44—C43	120.5 (5)
C5—C4—H7	109.1	C45—C44—H43	119.7
P3—C4—H7	109.1	C43—C44—H43	119.7
C5—C4—H8	109.1	C46—C45—C44	120.7 (5)
P3—C4—H8	109.1	C46—C45—H44	119.7
H7—C4—H8	107.8	C44—C45—H44	119.7
C6—C5—C4	112.2 (4)	C45—C46—C47	119.7 (5)
C6—C5—H9	109.2	C45—C46—H45	120.2
C4—C5—H9	109.2	C47—C46—H45	120.2
C6—C5—H10	109.2	C46—C47—C48	120.4 (5)
C4—C5—H10	109.2	C46—C47—H46	119.8
H9—C5—H10	107.9	C48—C47—H46	119.8
C5—C6—P4	111.6 (3)	C43—C48—C47	120.4 (5)
C5—C6—H11	109.3	C43—C48—H47	119.8
P4—C6—H11	109.3	C47—C48—H47	119.8
C5—C6—H12	109.3	C54—C49—C50	119.4 (5)
P4—C6—H12	109.3	C54—C49—P4	118.5 (4)
H11—C6—H12	108.0	C50—C49—P4	122.1 (4)
C8—C7—C12	120 (4)	C51—C50—C49	118.5 (5)
C8—C7—P1	119 (3)	C51—C50—H48	120.8
C12—C7—P1	121 (3)	C49—C50—H48	120.8
C7—C8—C9	119 (2)	C52—C51—C50	121.0 (5)
C7—C8—H13	120.4	C52—C51—H49	119.5
C9—C8—H13	120.4	C50—C51—H49	119.5
C10—C9—C8	119 (2)	C51—C52—C53	120.1 (5)
C10—C9—H14	120.4	C51—C52—H50	120.0
C8—C9—H14	120.4	C53—C52—H50	120.0
C11—C10—C9	121 (2)	C54—C53—C52	119.9 (5)
C11—C10—H15	119.5	C54—C53—H51	120.0
C9—C10—H15	119.5	C52—C53—H51	120.0
C10—C11—C12	120.6 (17)	C53—C54—C49	121.0 (5)
C10—C11—H16	119.7	C53—C54—H52	119.5
C12—C11—H16	119.7	C49—C54—H52	119.5
C7—C12—C11	120 (3)	C60—C55—C56	119.7 (5)
C7—C12—H17	120.0	C60—C55—P4	119.3 (4)
C11—C12—H17	120.0	C56—C55—P4	120.9 (4)
C18—C13—C14	117 (3)	C57—C56—C55	119.7 (6)
C18—C13—P1	121 (2)	C57—C56—H53	120.2
C14—C13—P1	121 (2)	C55—C56—H53	120.2
C15—C14—C13	121.8 (18)	C58—C57—C56	120.8 (6)
C15—C14—H18	119.1	C58—C57—H54	119.6
C13—C14—H18	119.1	C56—C57—H54	119.6
C14—C15—C16	116.9 (19)	C57—C58—C59	120.7 (6)
C14—C15—H19	121.5	C57—C58—H55	119.7
C16—C15—H19	121.5	C59—C58—H55	119.7
C17—C16—C15	122 (2)	C58—C59—C60	119.4 (6)
C17—C16—H20	119.0	C58—C59—H56	120.3
C15—C16—H20	119.0	C60—C59—H56	120.3
C16—C17—C18	119.7 (15)	C55—C60—C59	119.7 (6)
C16—C17—H21	120.1	C55—C60—H57	120.1
C18—C17—H21	120.1	C59—C60—H57	120.1
C13—C18—C17	122 (2)	Cl4—Ni1—Cl2	104.33 (6)
C13—C18—H22	118.9	Cl4—Ni1—Cl1	106.27 (6)
C17—C18—H22	118.9	Cl2—Ni1—Cl1	104.24 (7)
C24—C19—C20	119.1 (5)	Cl4—Ni1—Cl3	124.65 (6)
C24—C19—P1	122.6 (4)	Cl2—Ni1—Cl3	109.20 (6)
C20—C19—P1	118.3 (4)	Cl1—Ni1—Cl3	106.45 (6)
C21—C20—C19	119.8 (5)	C61—O1—C63	116.0 (8)
C21—C20—H23	120.1	O1—C61—C62	113.0 (9)
C19—C20—H23	120.1	O1—C61—H58	109.0
C22—C21—C20	120.4 (6)	C62—C61—H58	109.0
C22—C21—H24	119.8	O1—C61—H59	109.0
C20—C21—H24	119.8	C62—C61—H59	109.0
C23—C22—C21	119.7 (5)	H58—C61—H59	107.8
C23—C22—H25	120.2	C61—C62—H60	109.5
C21—C22—H25	120.2	C61—C62—H61	109.5
C22—C23—C24	121.2 (6)	H60—C62—H61	109.5
C22—C23—H26	119.4	C61—C62—H62	109.5
C24—C23—H26	119.4	H60—C62—H62	109.5
C23—C24—C19	119.8 (5)	H61—C62—H62	109.5
C23—C24—H27	120.1	O1—C63—C64	109.2 (9)
C19—C24—H27	120.1	O1—C63—H63	109.8
C30—C25—C26	119.5 (5)	C64—C63—H63	109.8
C30—C25—P2	118.2 (4)	O1—C63—H64	109.8
C26—C25—P2	122.4 (4)	C64—C63—H64	109.8
C25—C26—C27	119.7 (6)	H63—C63—H64	108.3
C25—C26—H28	120.1	C63—C64—H65	109.5
C27—C26—H28	120.1	C63—C64—H66	109.5
C28—C27—C26	119.9 (6)	H65—C64—H66	109.5
C28—C27—H29	120.1	C63—C64—H67	109.5
C26—C27—H29	120.1	H65—C64—H67	109.5
C29—C28—C27	120.1 (6)	H66—C64—H67	109.5
			
C13—P1—C1—C2	−175.8 (10)	C26—C25—C30—C29	−1.1 (9)
C19—P1—C1—C2	−64.6 (4)	P2—C25—C30—C29	178.4 (5)
C7—P1—C1—C2	−177.7 (13)	C25—P2—C31—C32	−91.2 (5)
Au1—P1—C1—C2	57.3 (4)	C3—P2—C31—C32	22.5 (5)
P1—C1—C2—C3	−175.6 (3)	Au2—P2—C31—C32	143.8 (4)
C1—C2—C3—P2	−163.2 (3)	C25—P2—C31—C36	95.1 (4)
C25—P2—C3—C2	−177.4 (3)	C3—P2—C31—C36	−151.1 (4)
C31—P2—C3—C2	67.7 (4)	Au2—P2—C31—C36	−29.9 (4)
Au2—P2—C3—C2	−55.2 (4)	C36—C31—C32—C33	−0.4 (8)
C43—P3—C4—C5	−173.8 (3)	P2—C31—C32—C33	−174.2 (4)
C37—P3—C4—C5	73.4 (4)	C31—C32—C33—C34	0.9 (9)
Au1—P3—C4—C5	−51.4 (4)	C32—C33—C34—C35	−1.9 (10)
P3—C4—C5—C6	−177.7 (3)	C33—C34—C35—C36	2.4 (9)
C4—C5—C6—P4	−162.3 (4)	C34—C35—C36—C31	−2.0 (9)
C49—P4—C6—C5	−67.3 (4)	C32—C31—C36—C35	1.0 (8)
C55—P4—C6—C5	−178.5 (4)	P2—C31—C36—C35	175.0 (4)
Au2—P4—C6—C5	57.1 (4)	C43—P3—C37—C42	−58.1 (4)
C19—P1—C7—C8	−67 (2)	C4—P3—C37—C42	54.6 (4)
C1—P1—C7—C8	47 (2)	Au1—P3—C37—C42	−179.1 (3)
Au1—P1—C7—C8	166.4 (17)	C43—P3—C37—C38	123.6 (4)
C19—P1—C7—C12	108 (2)	C4—P3—C37—C38	−123.8 (4)
C1—P1—C7—C12	−138 (2)	Au1—P3—C37—C38	2.5 (4)
Au1—P1—C7—C12	−18 (3)	C42—C37—C38—C39	−0.9 (8)
C12—C7—C8—C9	1 (3)	P3—C37—C38—C39	177.4 (4)
P1—C7—C8—C9	176.4 (18)	C37—C38—C39—C40	0.7 (8)
C7—C8—C9—C10	−3 (2)	C38—C39—C40—C41	−1.0 (9)
C8—C9—C10—C11	5 (4)	C39—C40—C41—C42	1.4 (9)
C9—C10—C11—C12	−6 (4)	C38—C37—C42—C41	1.4 (8)
C8—C7—C12—C11	−1 (4)	P3—C37—C42—C41	−177.0 (4)
P1—C7—C12—C11	−176.9 (16)	C40—C41—C42—C37	−1.6 (9)
C10—C11—C12—C7	4 (3)	C37—P3—C43—C48	141.5 (4)
C19—P1—C13—C18	146.0 (17)	C4—P3—C43—C48	29.6 (5)
C1—P1—C13—C18	−101 (2)	Au1—P3—C43—C48	−95.9 (4)
C7—P1—C13—C18	40 (31)	C37—P3—C43—C44	−46.8 (4)
Au1—P1—C13—C18	18 (2)	C4—P3—C43—C44	−158.7 (4)
C19—P1—C13—C14	−43.2 (19)	Au1—P3—C43—C44	75.8 (4)
C1—P1—C13—C14	69.5 (16)	C48—C43—C44—C45	0.2 (7)
Au1—P1—C13—C14	−171.1 (13)	P3—C43—C44—C45	−171.8 (4)
C18—C13—C14—C15	−5 (2)	C43—C44—C45—C46	−0.6 (8)
P1—C13—C14—C15	−176.5 (17)	C44—C45—C46—C47	0.0 (8)
C13—C14—C15—C16	2 (2)	C45—C46—C47—C48	1.0 (8)
C14—C15—C16—C17	2 (3)	C44—C43—C48—C47	0.8 (7)
C15—C16—C17—C18	−2 (3)	P3—C43—C48—C47	172.5 (4)
C14—C13—C18—C17	5 (3)	C46—C47—C48—C43	−1.4 (7)
P1—C13—C18—C17	176.5 (14)	C6—P4—C49—C54	155.1 (4)
C16—C17—C18—C13	−2 (2)	C55—P4—C49—C54	−89.9 (4)
C13—P1—C19—C24	85.3 (12)	Au2—P4—C49—C54	31.6 (4)
C1—P1—C19—C24	−25.6 (5)	C6—P4—C49—C50	−26.1 (5)
C7—P1—C19—C24	88.2 (15)	C55—P4—C49—C50	88.8 (4)
Au1—P1—C19—C24	−143.6 (4)	Au2—P4—C49—C50	−149.6 (4)
C13—P1—C19—C20	−94.0 (12)	C54—C49—C50—C51	1.1 (7)
C1—P1—C19—C20	155.1 (4)	P4—C49—C50—C51	−177.7 (4)
C7—P1—C19—C20	−91.0 (15)	C49—C50—C51—C52	−0.6 (8)
Au1—P1—C19—C20	37.1 (4)	C50—C51—C52—C53	0.6 (8)
C24—C19—C20—C21	−0.6 (8)	C51—C52—C53—C54	−1.2 (8)
P1—C19—C20—C21	178.7 (4)	C52—C53—C54—C49	1.7 (8)
C19—C20—C21—C22	1.1 (8)	C50—C49—C54—C53	−1.7 (7)
C20—C21—C22—C23	−1.5 (9)	P4—C49—C54—C53	177.1 (4)
C21—C22—C23—C24	1.4 (9)	C49—P4—C55—C60	92.3 (5)
C22—C23—C24—C19	−0.9 (9)	C6—P4—C55—C60	−153.6 (5)
C20—C19—C24—C23	0.5 (8)	Au2—P4—C55—C60	−29.7 (5)
P1—C19—C24—C23	−178.7 (4)	C49—P4—C55—C56	−83.7 (5)
C31—P2—C25—C30	−167.5 (5)	C6—P4—C55—C56	30.4 (5)
C3—P2—C25—C30	79.4 (5)	Au2—P4—C55—C56	154.3 (4)
Au2—P2—C25—C30	−41.8 (5)	C60—C55—C56—C57	−0.7 (9)
C31—P2—C25—C26	12.0 (5)	P4—C55—C56—C57	175.3 (5)
C3—P2—C25—C26	−101.1 (5)	C55—C56—C57—C58	−0.4 (9)
Au2—P2—C25—C26	137.7 (4)	C56—C57—C58—C59	0.7 (11)
C30—C25—C26—C27	2.1 (8)	C57—C58—C59—C60	0.0 (11)
P2—C25—C26—C27	−177.4 (4)	C56—C55—C60—C59	1.4 (9)
C25—C26—C27—C28	−2.2 (9)	P4—C55—C60—C59	−174.6 (5)
C26—C27—C28—C29	1.3 (11)	C58—C59—C60—C55	−1.1 (10)
C27—C28—C29—C30	−0.2 (12)	C63—O1—C61—C62	−178.7 (9)
C28—C29—C30—C25	0.1 (11)	C61—O1—C63—C64	−171.8 (9)
